# Pretemporal transcavernous transtentorial approach for right pontine cavernous malformation

**DOI:** 10.3171/2019.7.FocusVid.19156

**Published:** 2019-07-01

**Authors:** Xavier T. J. Hsu, Chih-Hsiang Liao, Chun-Fu Lin, Sanford P. C. Hsu

**Affiliations:** 1 Division of General Neurosurgery, Neurological Institute, Taipei Veterans General Hospital, Taipei;; 2 Department of Neurosurgery, Neurological Institute, Taichung Veterans General Hospital, Taichung;; 3 Institute of Medicine, Chung Shan Medical University, Taichung; and; 4 School of Medicine, National Yang-Ming University, Taipei, Taiwan

**Keywords:** cavernous malformation, Meckel’s cave, pretemporal, transcavernous, transtentorial, video

## Abstract

A 57-year-old man presented with acute changes in mental status. Brain CT showed a high-density lesion at the pons. Brain MRA revealed a very slow-flow vascular lesion at the right aspect of the pons, about 3.9 ⋅ 3.0 ⋅ 3.0 cm^3^, compatible with a pontine cavernous malformation (CM). Gross-total removal was achieved. In this approach, a wider surgical corridor was obtained by opening the Meckel’s cave and cutting the tentorium. For a midline attack point on the pons, additional removal of the posterior clinoid process can meet the goal. In the authors’ opinion, this approach is safe and effective in selected ventrolateral pontine CMs.

The video can be found here: https://youtu.be/moHqEkp5eCA.

## Transcript

Pretemporal transcavernous transtentorial approach for right pontine cavernous malformation. This is a 57-year-old man with progressive headaches, nausea/vomiting, drowsiness, and unsteady gait for 5 days. The brain CT showed a heterogeneous high-density lesion at the right aspect of the pons. The MR study was compatible with a cavernous malformation with recent intralesional hemorrhage at right side pons. The corticospinal tracts were pushed backwards by the cavernous malformation. Under general anesthesia, the patient was in supine position. The head was fixed by Mayfield head holder. A standard pterional craniotomy was performed with zygoma fracture. Flatten the sphenoid wing. Expose the periorbita. Peel the lateral wall of the cavernous sinus. Inject tissue glues for hemostasis. Remove the ACP extradurally. Open the Meckel’s cave. Cut the dura along the tentorium horizontally. Open the Liliequist membrane. Expose the fourth nerve. A vertical cut at the tentorium. Connect the vertical and horizontal cuts to release the tentorium. Intraoperative neuromonitoring: subcortical motor tract mapping. A small cortical incision was made. Open the lamina terminalis. The patient had transient third and fourth nerve palsies. Temporary worsening of dysmetria and muscle power of left limbs. In a pretemporal transcavernous transtentorial approach, we open the Meckel’s cave and cut the tentorium to get wider surgical corridor. This approach is effective for cavernous malformation located at the ventrolateral pons.

### Time points

00:25 Clinical vignette
00:30 PE/NE
00:35 Brain CT
00:40 Brain MR
00:55 Diffusion tensor imaging of corticospinal tracts
01:00 Diagnosis and approach
01:05 3D simulation of the surgical corridor
01:25 Patient positioning
01:30 Operation
06:10 Pathology report
06:15 Post-OP neurological status
06:23 Follow-up MRI
06:28 Neurological status at 2-year follow-up
06:38 Discussion
06:52 References
